# Notifications to Improve Engagement With an Alcohol Reduction App: Protocol for a Micro-Randomized Trial

**DOI:** 10.2196/18690

**Published:** 2020-08-07

**Authors:** Lauren Bell, Claire Garnett, Tianchen Qian, Olga Perski, Henry W W Potts, Elizabeth Williamson

**Affiliations:** 1 Department of Medical Statistics London School of Hygiene and Tropical Medicine London United Kingdom; 2 Research Department of Behavioural Science and Health University College London London United Kingdom; 3 Department of Statistics Harvard University Cambridge, MA United States; 4 Institute of Health Informatics University College London London United Kingdom; 5 Health Data Research UK London United Kingdom

**Keywords:** mobile health, digital behavior change, engagement, micro-randomized trial, push notifications, excessive alcohol consumption, smartphone app, alcohol, mHealth

## Abstract

**Background:**

*Drink Less* is a behavior change app that aims to help users in the general adult population reduce hazardous and harmful alcohol consumption. The app includes a daily push notification, delivered at 11 am, asking users to “Please complete your mood and drinking diaries.” Previous analysis of *Drink Less* engagement data suggests the current notification strongly influences how users engage with the app in the subsequent hour. To exploit a potential increase of vulnerability of excess drinking and opportunity to engage with the app in the evenings, we changed the delivery time from 11 am to 8 pm. We now aim to further optimise the content and sequence of notifications, testing 30 new evidence-informed notifications targeting the user’s perceived usefulness of the app.

**Objective:**

The primary objective is to assess whether sending a notification at 8 pm increases behavioral engagement (opening the app) in the subsequent hour. Secondary objectives include comparing the effect of the new bank of messages with the standard message and effect moderation over time. We also aim to more generally understand the role notifications have on the overall duration, depth, and frequency of engagement with *Drink Less* over the first 30 days after download.

**Methods:**

This is a protocol for a micro-randomized trial with two additional parallel arms. Inclusion criteria are *Drink Less* users who (1) consent to participate in the trial; (2) self-report a baseline Alcohol Use Disorders Identification Test score of 8 or above; (3) reside in the United Kingdom; (4) age ≥18 years and; (5) report interest in drinking less alcohol. In the micro-randomized trial, participants will be randomized daily at 8 pm to receive no notification, a notification with text from the new message bank, or the standard message. The primary outcome is the time-varying, binary outcome of *“Did the user open the app in the hour from 8 pm to 9 pm?”*. The primary analysis will estimate the marginal relative risk for the notifications using an estimator developed for micro-randomized trials with binary outcomes. Participants randomized to the parallel arms will receive no notifications (Secondary Arm A), or the standard notification delivered daily at 11 am (Secondary Arm B) over 30 days, allowing the comparison of overall engagement between different notification delivery strategies.

**Results:**

Approval was granted by the University College of London’s Departmental Research Ethics Committee (CEHP/2016/556) on October 11, 2019, and The London School of Hygiene and Tropical Medicine Interventions Research Ethics Committee (17929) on November 27, 2019. Recruitment began on January 2, 2020, and is ongoing.

**Conclusions:**

Understanding how push notifications may impact engagement with a behavior change app can lead to further improvements in engagement, and ultimately help users reduce their alcohol consumption. This understanding may also be generalizable to other apps that target a variety of behavior changes.

**International Registered Report Identifier (IRRID):**

DERR1-10.2196/18690

## Introduction

Excessive alcohol consumption inflicts an array of harms, causing various mental and physical illnesses, loss of productivity, and an increase in violence and traffic accidents [1,2]. There remains a large gap in delivering interventions to at-risk individuals despite the availability of screening and effective interventions [3,4]. As many as four out of five heavy drinkers who attend primary care do not receive screening and brief interventions [5], and this is in part due to barriers to large-scale implementation [6,7]. These barriers include time pressures within general practice, as well as a lack of support and training to successfully shift the consultation from treating the main presenting condition to offering a screening opportunity for hazardous drinking [8].

Behavior change apps promise to reduce this gap by providing real-time data capture and interventions [9-11]. Such behavior change apps, which aim to reduce excessive alcohol consumption, build onto a large body of literature demonstrating the effectiveness of text messages [12-15]. Similar to text messages, behavior change apps can be delivered at a low incremental cost per additional user (high scalability) and offer support to users in real-time. In addition, behavior change apps have the ability to gather data on users, thus enabling them to learn and evolve to become personalized to each individual user. However, a prime challenge for many behavior change apps is poor levels of engagement, with the frequency (number of sessions) and amount (time spent per session) sharply declining over time for the majority of users [16-18].

Engagement with a behavior change app can be considered in two dimensions, behavioral engagement, which can be measured as the amount, frequency, duration, and depth of use, and experiential engagement, characterized by attention, interest, and affect [19]. The behavioral aspect of engagement can be objectively measured through app use data. The effectiveness of a behavior change app can be moderated by a user’s engagement with the intervention’s active ingredients, and engagement fluctuates within and across users over time [20,21]. A push notification is a message that pops up on the phone, and may also vibrate, make a sound, or lock the screen to gain the user’s attention. Push notifications can be sent as a feature to enhance engagement and effectiveness by directing users to engage with the intervention’s modules when users likely need it the most [22]. The message can provide a connection between the moments of “point of care” when a user seeks an assessment and intervention, and “point of choice” when a user makes the decision to drink or not to drink [10,23,24].

*Drink Less* is a behavior change app for the general population of adults seeking to reduce hazardous and harmful alcohol consumption. The research and development of *Drink Less* has been described elsewhere [25-28]. Currently, users receive a daily push notification at 11 am, asking them to “Please complete your mood and drinks diary.” Following the SPIRIT (Standard Protocol Items: Recommendations for Interventional Trials) guidelines [29], the protocol reports our design for a micro-randomized trial (MRT) that aims to improve behavioral engagement (ie, frequency and amount of use) with *Drink Less* through the push notification.

### MRT as an Experimental Design for Optimizing Behavior Change Apps

The MRT design is a useful trial design for optimizing the *timing, content,* and *sequencing* for push notifications in a behavior change app [30-34]. During an MRT, individuals are repeatedly randomized to actionable notifications, or no notification, at prespecified decision points (Textbox 1). Along with longitudinally measuring a near-term outcome after each decision point, covariate data provided by wearables, sensors, or self-report may also be continuously gathered. Data evolves as a collection of time-varying covariates, treatments, and outcomes. A distinguishing feature of the MRT, compared to a parallel-group randomized controlled trial (RCT), is the repeated randomization over time. This repeated randomization aids further causal inferences that cannot be made when undertaking a parallel-group RCT.

**What does the repeated randomization of notifications in a micro-randomized trial (MRT) offer?**

MRT is an experimental design that provides information for developing more optimized policies or decision rules for delivering notifications. The repeated randomization within each individual in an MRT, which is absent in a parallel-group RCT, allows us to understand:

- If the notifications have a near-term effect on engagement, averaging over (i) the course of the study, (ii) all individuals, and (iii) the time-varying contexts individuals experience during the study.
- If the near-term effect of the notifications changes over time or depends on other time-varying covariates of the individual.
- If the notifications have a long-term effect on engagement, in addition to the possible near-term effect.

By understanding the above, researchers can build a more effective and less burdensome policy for delivering notifications, in order to improve users’ engagement with a behavior change app [35].

### The Drink Less App

*Drink Less* is a stand-alone app to help people reduce hazardous and harmful alcohol consumption [25-28]. The app was developed in line with the Multiphase Optimisation Strategy Framework [36] and the United Kingdom (UK) Medical Research Council guidance on complex interventions [37]. *Drink Less* contains seven different modules based on behavior change theory and evidence. These modules are (1) Normative Feedback, which is personalized feedback on how an individual’s drinking behavior compares to the recommended drinking levels; (2) Goal Setting, which allows users to set weekly “drinking reduction” goals, with brief advice on setting achievable goals; (3) Cognitive bias retraining, delivered through a game which targets users’ automatic biases by avoiding cues of alcoholic drinks and approaching nonalcoholic drinks; (4) Self-monitoring and Feedback, which users monitor and reflect on their alcohol consumptions, along with their mood, productivity, sleep and progress on goals; and (5) Action Planning, in which users create plans for dealing with difficult drinking situations. As of January 2, 2020, two new modules were added: (1) Behavioral substitution, which promotes substitution of drinking with a neutral behavior; and (2) Information about Antecedents, which provides users with information about social and environmental situations and events, emotions and cognitions that reliably predict drinking.

*Drink Less* launched in 2016 and is freely available on iTunes. At onboarding, users are asked to report their age, gender, type of employment (nonmanual, manual, or other) and to complete the Alcohol Use Disorders Identification Test (AUDIT) score [38,39]. The AUDIT is a 10-item screening tool for assessing alcohol consumption that helps identify people who would benefit from reducing or ceasing drinking. Users are also asked why they are using the app (“interested in drinking less alcohol” or “just browsing”).

In the existing version of the app, a push notification is sent daily at 11 am asking users to “Please complete your mood and drinks diary.” Accessing the app through the notification opens up the app and prompts users to complete their mood and drinks diary.

### User Engagement With Drink Less

An Ecological Momentary Assessment study with *Drink Less* users found that establishing a daily routine is important for maintaining engagement and that the daily notification supports such routines [20]. This study also found that perceived usefulness of the app (the belief that using the app will help the user to achieve their goal(s) and an indicator of users’ reflective motivation to engage) was associated with increased engagement for some users. The push notification may hence be most effective in improving engagement if it (1) supports the establishment of a routine (being sent at a set time), and (2) motivates to use particular intervention modules.

We visually explored patterns of engagement among a sample of 19,233 existing users of *Drink Less*. Further details of these results are available elsewhere (manuscript submitted). Data analysis from this cohort showed four important findings: (1) use over time decreased, with 50% of users disengaging (no use for seven or more consecutive days) after 22 days since download; (2) the existing daily notification, delivered at 11 am, is likely to have the strongest effect of near-term engagement in the subsequent hour; (3) the breadth of engagement is poor, with 85% of sessions occurring within the “Self-monitoring and Feedback” module; and (4), outside the 11 am notification period, a natural maximum of both frequency and length of sessions appeared in the evenings.

If and how to intervene at “peak-risk” moments is a key research priority [9,10,40,41]. Evenings are a time of day when people with a history of harmful alcohol consumption are the most vulnerable to continued, harmful drinking [42]. Additionally, the visual exploration of engagement patterns over time suggests evenings are an acceptable and opportune moment to engage with *Drink Less*. We decided to exploit the potential increase in vulnerability, opportunity, and acceptability of users in the evenings [43] and to test the marginal effect on near-term engagement of a bank of 30 new push notifications (see Multimedia Appendix 1) delivered at 8 pm. The new messages aim to promote the benefit of using specific intervention modules by targeting users’ reflective motivation to use the app.

We will undertake an MRT, with a single decision point of 8 pm, to assess the marginal effect of the new notifications on near-term engagement—use of the app in the hour following the notification—compared with both no notification and to a notification using the existing wording “Please complete your mood and drinking diaries.” Within the MRT, we aim to balance the objectives of learning how to optimize the push notification strategy, with the need to trial a good quality app that does not annoy users. Generally, in an MRT, the risk of annoying users with too many notifications over time could be mitigated with lower randomization probabilities. However, there are two reasons why we chose a single decision point to randomize notifications (8 pm), and not test multiple decision points within the day. Firstly, a single decision point allows users to establish an important routine with *Drink Less*, and secondly, this avoids asking users, through the design of the trial, to “Please complete your mood and drinking diaries” more than once within the day.

In order to explore how notifications influence overall engagement, the MRT will be complemented by two parallel trial arms; users will receive the standard notification daily at 11 am in one arm and will receive no notification on any day in the other. The two parallel arms provide us with (1) a momentary assessment of how engagement with *Drink Less* evolves over time when no notification is provided and (2) an exchangeable sample to compare the current policy of delivering a fixed notification daily at 11 am, to randomly varying the content and sequence of notifications at 8 pm.

### Aims and Objectives

#### Aim

This study aims to assess the push notification strategy and to improve engagement with *Drink Less* during the first 30 days following download.

#### Primary Objective

The primary objective of the study is to estimate the marginal effect of a notification (pooling both types of messages, the standard wording and the new bank of messages) on near-term engagement, defined as the use of the app in the hour following the notification decision point (8 pm to 9 pm ).

#### Secondary Objectives

The secondary objectives are as follows:

1. Compare the marginal effect of the new bank of 30 messages to the standard wording of “Please complete your mood and drinking diary” on near-term engagement, defined as the use of the app in the hour following the notification decision point (8 pm to 9 pm).
2. Explore whether the effect of a notification (pooling both types of messages) on near-term engagement decreases over time.
3. Estimate the lagged effect of prior notifications on near-term engagement.
4. Understand how the notification effect is moderated by time-varying covariates (use before 8 pm, use on the previous day, weekend/weekday effect).
5. Investigate if the effect of the notifications depends on baseline characteristics (gender, age, employment type, AUDIT score).
6. Examine overall engagement during the 30 days following download in users receiving no notifications, those who receive the standard notification daily at 11 am, and those who receive a mix of notifications at 8 pm.

### Trial Design

This study is an MRT with two additional parallel arms. Multimedia Appendix 2 illustrates the participant flow through the trial. It also shows which outcomes will be obtained from either the MRT or the two additional trial arms.

The MRT will test the effect of both delivering standard message content and a bank of varied message content on near-term engagement, compared to receiving no message.

Sixty percent of eligible users will be randomly assigned to participate in the MRT. The remaining eligible users will be randomized in equal numbers to the two parallel arms of either receiving no notifications (Secondary Arm A) or daily notification of the standard message of *“Please complete your mood and drinking diary”* (Secondary Arm B).

Among users assigned to the MRT, every day at 8 pm (the “decision point”), each user will be randomized to receive one of three options: no notification, the standard message, or a notification selected at random from the bank of new messages. The randomization probabilities for the decision points each day are 40% to receive no notification, 30% to receive the standard message, and 30% to receive a randomly selected message from the bank of new messages.

## Methods

### Participants, Interventions, and Outcomes

#### Study Setting

*Drink Less* is freely available on the iTunes Store. This trial will recruit eligible new individuals who download the *Drink Less* app during the trial recruitment period, from January 2, 2020, to April 1, 2020 (app version: 2.0.1).

We extended the informed consent process for this trial to comply with ethics requirements. At onboarding, users will be first asked to read the privacy notice and participant information sheet, then provide informed consent (see Multimedia Appendices 2 and 3), before proceeding. Users who do not consent to take part in the research will be provided the standard version of the app.

#### Eligibility Criteria

Users who download *Drink Less* during the recruitment period will be eligible to participate if they: self-report a baseline Alcohol Use Disorders Identification Test (AUDIT) score of 8 or above, indicating excessive alcohol consumption [39,44]; reside in the UK; are aged 18 years or over, and report themselves to be interested in drinking less alcohol.

#### Intervention

A bank of 30 novel messages was developed with the aim of increasing users’ reflective motivation to engage with the app (see Multimedia Appendix 1). All messages contain the phrase “(using a particular module in the app) *can help you drink less*.” As perceived usefulness of the app has previously been found to be associated with increased engagement, we hypothesized that new messages which highlight the benefits of using the app would increase users’ reflective motivation to use the app and hence generate higher rates of engagement compared with the standard, existing message.

### Measures

Outcome measures for the MRT will be collected continuously over the 30 days following the download of *Drink Less*. These measures collected over time are: when users open the app, when each module is used, the length of time (seconds) spent on the app and drinking records, with the date of drinks consumed and date and time of records made. Outcomes for the wider comparison between the parallel trial arms are defined over the whole 30-day follow-up period.

### Outcomes

#### Primary Outcome (MRT)

The primary outcome measure in the MRT is a time-varying, binary proximal (ie, near-term) measure of engagement (use of the app). Specifically, the primary outcome for the MRT is whether the user opens the app in the hour (8 pm to 9 pm) following the randomization of receiving a notification at 8 pm.

#### Secondary Outcomes Collected Daily Through the Trial Period (MRT)

Within the MRT, the two secondary outcomes below will be defined in the hour following the decision point (8 pm to 9 pm):

1. whether or not the user creates an entry in their drink calendar (by either recording a drink record or recording a drink-free day);
2. the time, in seconds, spent on the app;

#### Secondary Outcomes Over the Whole Trial Period (MRT and Parallel Arms)

In the parallel trial arms, secondary outcomes that will be explored are:

1. the number of days to complete disengagement, defined as the first day of at least seven consecutive days of no use from day of download;
2. the total number of sessions over the 30 days following download;
3. the total time, in seconds, spent on the app over 30 days since download, overall, and by intervention module.

### Time-Varying Covariates

Measured covariates which vary over time within individuals are the use of the modules (Action Planning; Cognitive Bias Re-Training; Self-monitoring and Feedback; Behavioral Substitution; Goal Setting; Normative Feedback; Information About Antecedents); entry of drink (or alcohol-free) record in each session; if the user opened the app before 8 pm that day; and if the user opened the app the day before.

### Time-Fixed Covariates

Measured time-fixed covariates are age, gender, type of employment (manual, nonmanual, or other), day of the week of download, and baseline AUDIT score.

### Sample Size

We aim to randomly assign 1200 users to the MRT arm, 400 users to the standard daily notification arm (Secondary Arm A), and 400 users to the no notification arm (Secondary Arm B), resulting in a total of 2000 participants. The sample sizes were calculated as follows:

To estimate the sample size required for the MRT arm, we used a simulation-based approach to determine the sample size required to attain a prespecified power level, because currently there is no off-the-shelf software to calculate the sample size for MRTs with binary outcomes. Our primary objective is to understand the marginal effect of receiving a push notification at 8 pm on engagement, with an important secondary objective towards the tailoring of the notification policy is identifying effect moderation over time. Plausible estimates of a treatment effect and effect moderation were obtained by exploring patterns of use with *Drink Less*. With 80% power and 5% type I error, we have sized this trial to detect a marginal treatment effect of 2.16, which decays by a factor of 0.911 by day since download. This is close to 100% power for our primary objective, the marginal effect. See Multimedia Appendix 4 for more details.

The sample size of the two additional parallel arms was determined based on the secondary outcome of time to disengagement (no use for seven or more consecutive days). Analysis of the current app shows that 55% of users have disengaged by day 30. We powered this sample size based on a minimal relevant change in disengagement of 10%, such that we expect 65% of users to disengage by day 30 when no notifications are delivered. With a 5% type I error and 80% power to detect an increase in disengagement to 65% of users by day 30, we would require 372 users per arm. To simplify the allocation process, we rounded-up the sample size for the parallel arms to 400 users per arm each, resulting in an overall sample size of 2000 and an allocation ratio of 60% to the MRT and 20% to each parallel arm.

### Anticipated Recruitment Rate

The available recruitment window with the app was January 2, 2020, to April 1, 2020. All new app users who meet the eligibility criteria, provide consent, and complete app onboarding during this period will be recruited into the trial. Previous analyses of cohort data of existing users suggest that the average number of downloads by eligible users will be 33 per day, through the 59-day recruitment period. If the number of participants exceeds the minimum required sample stated above, we will continue to recruit until the end of the predefined recruitment period.

### Assignment of Interventions

#### Sequence Generation

At recruitment, 60% of participants will be randomized to the MRT. The remaining participants will be randomly allocated 50:50 to receive no notifications or to receive standard notifications daily at 11 am.

Among the participants randomized to the MRT, at 8 pm each participant will be randomized daily to receive one of three options: no notification, the standard notification wording, or a message randomly selected from the new message bank. The randomization probabilities for these three options will be 40%, 30%, and 30%, respectively.

The randomization probabilities are fixed across all individuals and do not depend on individuals’ time-varying treatment, outcomes, or covariates.

#### Allocation Concealment Mechanism

Users will be aware of whether or not they have received a push notification each day. They will be informed in the consent procedures that they are part of a research study testing how different versions of the app affect use. However, they will not receive explicit information that we are interested in the effect of the notification, about which arm of the study they have been allocated to, the full design of the study, or the planned schedule of their notifications. The standard request for users to enable notifications at the end of the onboarding process was disabled for this trial.  Users were still able to turn off the notifications through their phone settings.

#### Implementation

Simple randomization was used, with no stratification or blocking. The code to generate the randomization sequencing was developed and coded into the app by an external app developer. Members of the trial team verified the randomization process.

### Data Collection, Management, and Analysis

Descriptions of the trial participants, in terms of their available baseline data, will be reported for all MRT participants and participants in the two additional arms.

#### Primary Analysis (MRT)

Our primary analysis will estimate the marginal effect of the notifications on the binary, time-varying outcome of whether or not a user opens the app between 8 pm and 9 pm. The marginal effect is averaged over all days and all participants in the MRT arm.

The effect of notifications, quantified as a relative risk, with a 95% confidence interval, will be assessed using the estimator for marginal excursion effect for MRTs with binary outcomes [45]. The excursion effect is a causal effect concerning what would happen if an individual followed the notification scheduled used in the MRT up to day *t–*1 and then deviated from the schedule to receive a notification at day t, versus deviated from the schedule to receive no notification at day *t*. The notification schedule used in the MRT is the delivery of push notifications with 40%, 30%, 30% probability every evening at 8 pm (see the last paragraph of the subsection Trial Design). The marginal excursion effect we consider in the primary analysis will marginalize (ie, average) overall days and all individuals. Because the near-term outcome is binary, we will estimate the marginal excursion effect on the log relative risk scale.

For this analysis, both types of notification—the standard wording and the messages drawn from the new bank of messages—will be pooled; the comparison will be between any notification versus no notification. *P* values less than .05 will be considered statistically significant. Models used in the primary and secondary analyses for the MRT arm will adjust for age, gender, employment type, baseline AUDIT score, the number of days since download, if the user opened the app before 8 pm, and if the user opened the app the day before.

#### Secondary Analyses (MRT)

Our secondary analyses will assess the effect of sending a notification from the bank of 30 new messages compared to the standard message “Please complete your mood and drinking diaries” on the primary outcome; that is, whether the user opened the app between 8 pm and 9 pm. We will use the same analysis method here as for the primary analyses, which is the estimator for the marginal excursion effect. We will also assess the effect notifications have on users creating an entry to their drinks calendar.

We will investigate the effect moderation of the notification by day in the study, quantified as an interaction, and expressed as a relative risk. We will also examine the sensitivity of the result when day-in-study is replaced by splines or its log-transformation. Lagged notification effects will be similarly quantified.

The continuously valued secondary outcome in the MRT relating to time spent on the app (seconds) will be analyzed using a centered and weighted least-squares estimation method [46] with the effect quantified using the mean difference. All secondary outcomes will be explored by comparing any notification versus none and then separating the two types of notifications.

#### Secondary Analyses (Parallel Arms)

Time to complete disengagement will be analyzed using the Kaplan-Meier estimator. A Cox proportional hazards model will be used to estimate the hazard ratio for disengagement comparing the three parallel arms. The proportional hazards assumption will be assessed graphically and using tests based on Schoenfeld residuals. If nonproportionality is detected, methods allowing for this will be applied and presented as exploratory analyses alongside the previous Cox model analysis.

Linear regression models with robust standard errors will be used to compare the time spent on the app, both overall and on specific modules, between the three parallel trial arms. Similar models will be used to compare the total number of days of app use between arms.

No adjustment will be made for multiple testing. Outcomes and analyses are categorized by the degree of importance (primary and secondary), and results will be interpreted in the light of that ordering.

## Results

This study received funding from the MRC Network of Hubs for Trials Methodology Research (MR/L004933/2- R18) in January 2019. As of early March 2020, at the date of manuscript submission, the trial is ongoing, with 452 users recruited.

### Data Collection and Data Monitoring

Data collection began on January 2, 2020, and will end on May 1, 2020. Due to the rapid nature of this research, and relatively very low risk of adverse events due to the intervention, there will be no interim analysis or Data Monitoring during the trial.

### Ethics and Dissemination

Ethical approval was granted by the London School of Hygiene and Tropical Medicine Interventions Research Ethics Committee (17929) and the University College London Departmental Research Ethics Committee (CEHP/2016/556); an amendment was granted by the Ethics Amendment Request to Work Package One *“The application of digital technologies to advance the understanding, and improve the implementation of behavior change.”*

### Confidentiality

No identifiable data will be collected during this study.

## Discussion

The study will determine whether sending a notification at 8 pm increases engagement in the subsequent hour with *Drink Less* and whether the impact of the notification changes over time. Previous research has found that the perceived usefulness of the app is a predictor of both the amount and frequency of engagement with *Drink Less* [20]. Building on these findings, secondary analyses will systematically explore if messages which aim to increase the perceived usefulness of the app by encouraging users to try out various modules are more effective at increasing engagement than the standard request to record drinking and mood diary entries. We will also explore potential effect moderation, lagged effects, and overall summaries of use over 30 days since download. This study will provide evidence of how notifications affect engagement, as well as considerations towards further improvement of the push notification policy.

Our research is limited by the lack of outcomes to understand a change in alcohol units consumed, meaning we could not investigate whether receiving notifications had any effect on hazardous and harmful alcohol consumption. Generally, gathering valid and reliable health outcome measures over time, solely through self-reports, is a prime challenge for the digital health community [47,48]. *Drink Less* prompts users to complete the AUDIT-C one month after downloading the app, but the proportion of users to do so is low [49]. Our primary aim is to improve engagement with the app, and future research can investigate whether any effectiveness is mediated through engagement. Importantly, research into effective strategies to collect real-time outcomes on substance abuse through other apps is emerging [50-52], including an MRT with an “engagement-first” strategy to increase the rate of self-reported data [53]. This research is a valuable step towards developing more effective behavior change apps. Another limitation is that we do not understand if users subsequently turned off their notifications during the trial through their phone’s settings.

Methodologies for tailoring notification policies, either as a stratified intervention based on time-varying or time-invariant covariates (eg, day of the week, age, past moods, previous app use or drinks reported), or strictly personalized policies, in which user’s own responses to prior notifications inform the future policy, are becoming increasingly more refined [54,55]. After establishing whether there is a marginal effect of the push notifications and gaining a better understanding of the push notification’s role in the dynamic nature of engagement, subsequent studies may address the more ambitious aims of creating a sequence of decision rules. Such decision rules could capitalize on dynamic states of opportunities within users’ current environment or adapt to a user’s history. This may be achieved by better understanding how the between- and within-person effects of the notification [56] change under varying circumstances, as well as any lagged effects of notifications.

## Appendix

Multimedia Appendix 1 Bank of 30 newly developed messages and their link to the relevant behavior change module.

Multimedia Appendix 2 User flow chart for the micro-randomized trial with two additional parallel arms.

Multimedia Appendix 3 Privacy notice.

Multimedia Appendix 4 Information sheet.

Multimedia Appendix 5 Sample size calculation.

## Abbreviations

AUDIT: Alcohol Use Disorders Identification Test
MRT: micro-randomized trial
NIHR: National Institute for Health Research
RCT: randomized controlled trial
SPIRIT: Standard Protocol Items: Recommendations for Interventional Trials
