# Supplementary material for: Notifications to Improve Engagement With an Alcohol Reduction App: Protocol for a Micro-Randomized Trial
Source: JMIR Res Protoc. 2020 Aug 7;9(8):e18690. doi: 10.2196/18690 (PMC7442945; doi:10.2196/18690)
Supplement: Multimedia Appendix 1 [file resprot_v9i8e18690_app1.docx]

Bank of 30 newly developed messages and their link to the relevant behavior change module.

| Message content | Behavior change module |
| --- | --- |
| Tracking your drinks and days you don’t drink can help you drink less. | Goal Setting |
| Did you know that tracking your drinks and days you don’t drink can help you drink less? | Goal Setting |
| Tracking your drinks and days you don’t drink can help you drink less. Take a moment to track your drinks or a drink-free day. | Goal Setting |
| Setting a doable goal can help you drink less. | Action Planning |
| Did you know that setting a doable goal can help you drink less? | Action Planning |
| Setting a doable goal can help you drink less. Take a moment to set a doable goal. | Action Planning |
| Tracking your mood after drinking and drink-free days can help you drink less. | Self-Monitoring and Feedback |
| Did you know that tracking your mood after drinking and drink-free days can help you drink less? | Self-Monitoring and Feedback |
| Tracking your mood after drinking and drink-free days can help you drink less. Take a moment to track your mood. | Self-Monitoring and Feedback |
| Tracking your productivity levels after drinking and drink-free days can help you drink less. | Self-Monitoring and Feedback |
| Did you know that tracking your productivity levels after drinking and drink-free days can help you drink less? | Self-Monitoring and Feedback |
| Tracking your productivity levels after drinking and drink-free days can help you drink less. Take a moment to track your productivity levels. | Self-Monitoring and Feedback |
| Tracking your sleep quality after drinking and drink-free days can help you drink less. | Self-Monitoring and Feedback |
| Did you know that tracking your sleep quality after drinking and drink-free days can help you drink less? | Self-Monitoring and Feedback |
| Tracking your sleep quality after drinking and drink-free days can help you drink less. Take a moment to track your sleep quality. | Self-Monitoring and Feedback |
| Tracking how clear-headed you feel after drinking and drink-free days can help you drink less. | Self-Monitoring and Feedback |
| Did you know that tracking how clear-headed you feel after drinking and drink-free days can help you drink less? | Self-Monitoring and Feedback |
| Tracking how clear-headed you feel after drinking and drink-free days can help you drink less. Take a moment to track your clear-headedness. | Self-Monitoring and Feedback |
| Keeping an eye on how your drinking compares with others can help you drink less. | Normative Feedback |
| Did you know that keeping an eye on how your drinking compares with others can help you drink less? | Normative Feedback |
| Keeping an eye on how your drinking compares with others can help you drink less. Take a moment to check how your drinking compares with others. | Normative Feedback |
| Recording if-then plans can help you drink less. | Action Planning |
| Did you know that recording if-then plans can help you drink less? | Action Planning |
| Recording if-then plans can help you drink less. Take a moment to record an if-then plan. | Action Planning |
| Keeping an eye on which if-then plan has and hasn’t worked can help you drink less. | Action Planning |
| Did you know that keeping an eye on which if-then plan has and hasn’t worked can help you drink less? | Action Planning |
| Keeping an eye on which if-then plan has and hasn’t worked can help you drink less. Take a moment to check your if-then plans. | Action Planning |
| Playing the “yes please, no thanks” game can help you drink less. | Cognitive Bias Re-training |
| Did you know that playing the “yes please, no thanks” game can help you drink less? | Cognitive Bias Re-training |
| Playing the “yes please, no thanks” game can help you drink less. Take a moment to play the game. | Cognitive Bias Re-training |
