# Supplementary figures and images for: Notifications to Improve Engagement With an Alcohol Reduction App: Protocol for a Micro-Randomized Trial
Source: JMIR Res Protoc. 2020 Aug 7;9(8):e18690. doi: 10.2196/18690 (PMC7442945; doi:10.2196/18690)

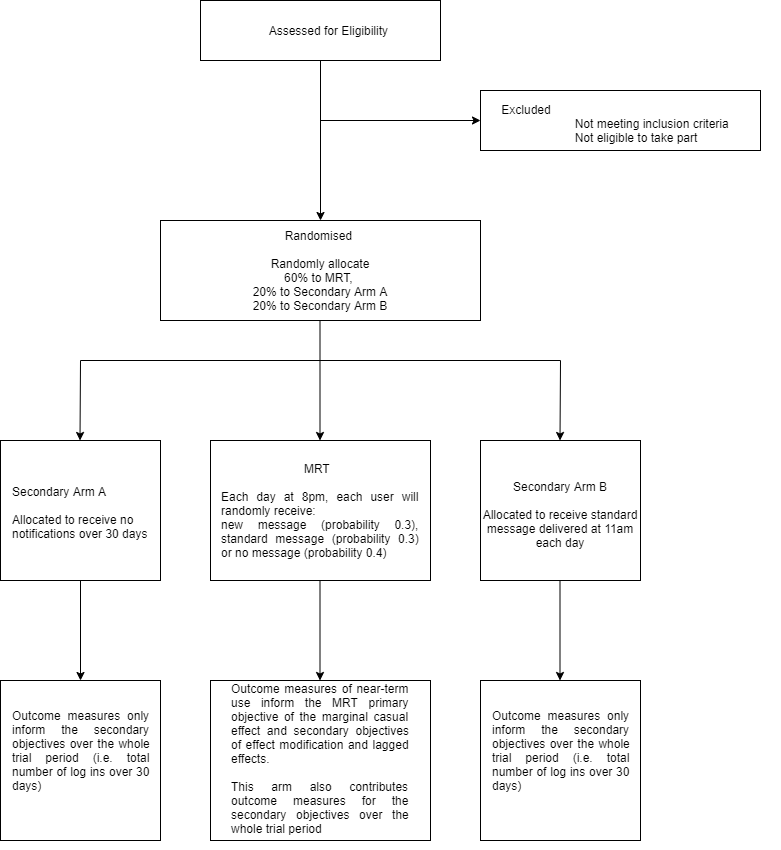

Supplement: Multimedia Appendix 2 [file resprot_v9i8e18690_app2.png]
