# Supplementary material for: Notifications to Improve Engagement With an Alcohol Reduction App: Protocol for a Micro-Randomized Trial
Source: JMIR Res Protoc. 2020 Aug 7;9(8):e18690. doi: 10.2196/18690 (PMC7442945; doi:10.2196/18690)
Supplement: Multimedia Appendix 3 [file resprot_v9i8e18690_app3.docx]

**Privacy notice**

The data we collect is anonymous and will only be used for academic research conducted by University College London to develop improved ways of helping people to drink less.

We are currently testing how the push notifications you receive affect how you use the app. In this research, you may be randomized to receive either no messages, the same message, or different messages over time.

The Tobacco and Alcohol Research Group at University College London (UCL) is collecting the data and processes users’ data for the following purposes:

• To help users drink less

• To improve the smartphone application

• To conduct research and write publications that add to the scientific literature

• To communicate with users

The data collected includes that which the user voluntarily enters or provides when using the Drink Less app (eg, gender, age, country, job type, alcohol consumption) and app usage. App versions up to and including 1.0.10 collected personal data—email addresses—which was entered voluntarily.

Those personal data will be stored for three years.

Processing of users’ data is necessary for the performance of a task carried out in the public interest. Health data on alcohol consumption is necessary for scientific research purposes and is in accordance with safeguards.

Research governance within UCL ensures that data is:

• Necessary to support research

• Only used to support legitimate research activities that are considered to be in the public interest

• Safeguarded/protected

Disclosure:

The Tobacco and Alcohol Research Group at UCL will not share user data with any third parties.

Right of access:

As the data collected is anonymous, it is not possible to link any data we hold with an individual user.

For versions up to and including 1.0.10 of the app, email addresses were collected from some users. If a user downloaded one of these app versions and entered their email address, then the Tobacco and Alcohol Research Group at UCL can share the data held on the user. Up to three years after downloading the app, users can request this by emailing [support@drinklessalcohol.com](mailto:support@drinklessalcohol.com), along with the email address entered during the registration process. The Tobacco and Alcohol Research Group at UCL will then send users the data that it holds on them within one calendar month of receipt.

If you have any questions about this privacy notice, please contact us at [support@drinklessalcohol.com](mailto:support@drinklessalcohol.com)

Terms and conditions

All data will be stored securely and in line with our privacy notice. You are not obliged to have your data used for academic research, and you should not feel coerced. If you choose to withdraw, you may do so without a disadvantage to yourself and without any obligation to give a reason. To withdraw, please go to the Help tab of the app and choose “Opt out of the study.”

Please feel free to ask us any questions on [support@drinklessalcohol.com](mailto:support@drinklessalcohol.com)

Consent

By consenting to this Privacy Notice, you are explicitly giving the Tobacco and Alcohol Research Group at UCL permission to process your data for the purposes specified.

You may withdraw consent at any time by going to the Help tab of the app and choosing “Opt out of the study.”

I consent to the use of my data, as explained by the Privacy Notice and Terms and Conditions.

[Yes, I agree]

[No, I disagree]
