# Supplementary material for: Notifications to Improve Engagement With an Alcohol Reduction App: Protocol for a Micro-Randomized Trial
Source: JMIR Res Protoc. 2020 Aug 7;9(8):e18690. doi: 10.2196/18690 (PMC7442945; doi:10.2196/18690)
Supplement: Multimedia Appendix 4 [file resprot_v9i8e18690_app4.docx]

**Information Sheet**

[Screen2] Information sheet for a research project

You are being invited to take part in a research project. Before you decided to take part, it is important for you to understand why the research is being done and what participation will involve. The information here will try and answer any questions you might have about the study but contact us at support@drinklessalcohol.com if there is anything else you would like to know. Please read the following carefully and discuss it with others if you wish.

*Who is conducting the research?*

We are a team of researchers at University College London and London School of Hygiene and Tropical Medicine.

*Why are we doing this research?*

We want Drink Less to be an app that you want to use. There are features in the app which may or may not encourage you to carry on using the app. We want to know how to improve these features. We are only interested in how you use the app during the first 30 days.

*Why have I been chosen?*

You are eligible to take part in this study if you are aged 18 years old or older, have indicated that you are interested in using the app for drinking less alcohol, and have a score of 8 or more on the test to identify alcohol use disorders, as we are particularly keen to support this group in continuing to use the app. Most importantly, you decide to take part or not.

*What will happen if I choose to take part?*

If you choose to take part in this study, the reminders that Drink Less sends you may have different wording compared with people who choose not to take part. Otherwise, your experience with the app will not be different from other people.

*Could there be problems for me if I take part?*

We do not anticipate any problems caused by taking part in this study.

*What data will you collect about me?*

We will not at any time access any personal information about you, such as your name, address, email address. We will collect data about your age, gender, occupation type, and your alcohol consumption when you download the app. For the following 30 days, we will collect data about how you use the app, such as did you use the app or how long you spent on the app. We cannot identify you in this study, and the app will not provide us with any other information available on your phone.

*What will happen to my data at the end of the research?*

At the end of the study, we will make a dataset available to the public here: https://osf.io/q8mua. It will not be possible for anyone to identify you in the dataset. The dataset will only contain your general characteristics (age, gender, occupation type, baseline alcohol consumption) and how you used the app for 30 days, such as did you use the app or how long you spent on the app. The content of the diaries will not be made available to us or anyone else.

If you have any questions, please feel free to ask Professor Susan Michie on support@drinklessalcohol.com

*Consent*

You are about to consent to a scientific study that examines how people use this app.

By taking part, you agree that you have read and understood the information about the study.

By consenting to this Privacy Notice, you are explicitly giving the Tobacco and Alcohol Research Group at UCL permission to process your data for the purposes specified.

I consent to my data being fully anonymized and made available in the public domain via a data repository. **Tick Box** [Yes, I agree] [No, I disagree]

I understand that it is not possible to identify me at any stage in this research, or from any information made public. **Tick Box** [Yes, I agree] [No, I disagree]

I understand I may withdraw consent at any time by going to the Help tab of the app and choosing “Opt out of the study.” **Tick Box** [Yes, I agree] [No, I disagree]

I consent to the use of my data, as explained by the Privacy Notice and Terms and Conditions.

[Yes, I agree]

[No, I disagree]
